# Characterizing a community health partnership in Dominican Republic: Network mapping and analysis of stakeholder perceptions

**Published:** 2018-05-31

**Authors:** Kristy Yiu, Helen Dimaras, Olga Valdman, Bido Franklin, John Prochaska, Lawrence Loh

**Affiliations:** 1 McMaster University, Ontario, Canada; 2 University of Toronto, Department of Ophthalmology & Vision Sciences, Faculty of Medicine, Ontario, Canada; 3 University of Toronto, Division of Clinical Public Health, Dalla Lana School of Public Health, Ontario, Canada; 4 The Hospital for Sick Children: Department of Ophthalmology & Vision Sciences; Child Health Evaluation Sciences Program, Ontario, Canada; 5 University of Massachusetts Medical School, Massachusetts, United States; 6 Hospital El Buen Samaritano, La Romana, Dominican Republic; 7 University of Texas Medical Branch, Department of Preventative Medicine & Community Health, Texas, United States; 8 The 53^rd^ Week, New York, United States

## Abstract

**Background:**

Medical trainees complete learning experiences abroad to fulfil global health curricular elements, but this participation has been steadily criticized as fulfilling learner objectives at the cost of host communities. This study uses network and qualitative analyses in characterizing a community coalition in order to better understand its various dimensions and to explore the perceived benefits it provided towards optimizing community outcomes.

**Methods:**

Data from a semi-structured survey was used for network and qualitative analyses. Partner linkages were assessed using network analysis tool UCINET 6 (version 6.6). Thematic analysis was conducted on qualitative responses around the perceived coalition strengths and weaknesses.

**Results:**

Network analysis confirmed that local member organizations were key network influencers based on reported formal agreements, general interactions, and information shared. While sharing of resources was rare, qualitative analysis suggested that information sharing contributed to engagement, enthusiasm, and communication that allowed visiting partners to expand their understanding of community needs and shift their focus beyond learner objectives.

**Conclusion:**

Global health programs for medical students should consider the use of community health coalitions to optimally align the work undertaken by learners on global health experiences abroad. Network mapping can help educators and coalition partners visualize interactions and identify value.

## Introduction

An increasing number of medical students and faculty educators are participating in educational experiences abroad to fulfil academic requirements in the global health curriculum.^1,2^ The popularity of such training has paralleled the growth of volunteer abroad opportunities in high-income countries, driven by study abroad programs offered by academic institutions, charity work organized by faith-based or community groups, and “voluntourism” experiences promoted by both non-profit and for-profit organizations.^1,3^

Established literature has documented concerns that such experiences provide notable benefits to learners and institutions while imposing burdens on host communities abroad.^4-6^ Described burdens include misalignment between local priority needs and educational program goals, potential disruption of the local economy, harms from clinical services provided (particularly by trainees), lack of continuity and follow-up, “cultural colonialism,” and the development of dependence on educational and volunteer teams.^5,6^ One commonly cited reason for many of these burdens is little to no local partnership and guidance in the conduct of such educational experiences.^7^ Critics have in fact stated that such experiences sometimes even purport to “provide developmental aid” when in reality, the goal is for students to travel and gain international experience.^8^

In this study, we use network mapping to demonstrate that a healthy community partnership with the right actors and agents involved can help resolve some of these concerns by providing channels by which to align local priorities with potential inputs and impacts of visiting educational groups. We further note that the use of network analysis and other partnership assessment tools might help educational institutions characterize and strengthen the partnerships that they become involved in.

The setting of the study is La Romana, Dominican Republic, a community that has seen a growing number of visiting groups since the 1980s that have participated in short-term health and development programs for educational and volunteer purposes. These efforts have been targeted at migrant Haitian populations in the area that face systemic societal discrimination and subsequent limitations to health and social services. Many visiting medical trainees and faculty have attempted to address these gaps during their educational visits to the community but continuing concerns exist around the sustainability and appropriateness of their work.^9^

The *Coalicion de Salud Comunitaria* (Community Health Coalition, COSACO) was developed in March 2015 in an attempt to address the concerns associated with short-term volunteer and educational groups working in La Romana. Based on a modified Healthy Community Partnership model, COSACO brings together traditional partners such as local health and healthcare stakeholders (e.g., the local health department and key local health facilities) with non-traditional partners that indirectly influence health and development efforts (e.g., local government, private enterprise, and academia).^10^ The specific modification is that visiting short-term volunteer groups, including educational endeavours, are also included.^11^ The mission of COSACO is to connect traditional and non-traditional partners thus providing them with an opportunity to align their efforts in optimizing health and providing health care services to the migrant Haitian population in the Dominican Republic.

Unlike traditional stand-alone global health educational program models that do not involve a broad base of local stakeholders, we hypothesized that COSACO could optimize community outcomes from visiting educational efforts by aligning the work of trainees with locally defined community health and development goals. Presumably this occurs through the partnerships that are developed between visiting academic institutions and key local stakeholders, thereby resulting in increased information sharing and joint project and priority development.

To better understand the framework by which COSACO operates to promote these benefits, we employed network analysis to characterize the various dimensions of the coalition around information and resource sharing, and formal agreements that would otherwise not exist in the coalition’s absence.^12^ We then supplemented this characterization with a qualitative analysis that provided additional detail into perceived benefits that the coalition provided towards optimizing the outcomes of visiting educational efforts.

## Methods

### Study design

This was a cross-sectional mixed-methods study. COSACO partners were invited to complete a semi-structured survey (Appendix A) provided in both English and Spanish. These responses were subsequently analyzed in two phases. First, we completed a network analysis to map out existing network patterns of relationships between partners. Second, we produced a qualitative thematic analysis on described perceptions around the a) characteristics of those partnerships and b) how they might contribute to optimizing the outcomes of visiting educational endeavours.^13^

The study was approved by the University of Toronto Health Sciences Research Ethics Board (protocol #32378).

### Survey procedures

Recruitment for this study employed a fixed list sample methodology, based on the predefined network of partner members within the coalition. The 13 COSACO members were invited to participate either via e-mail or during an in-person meeting conducted over a one-week field visit of the research team to La Romana. Follow-up emails were sent to encourage survey completion as well as to address responses that were incomplete or unclear upon the review of returned surveys. None of the surveys returned were partially completed.

### Survey measures

Survey questions aimed to review four broad aspects of the network: general information of the participating partner agency, evaluation of that agency’s interactions with other COSACO partners, general satisfaction with the partnerships, and perceptions of existing partnerships (Appendix A).

The first section of the survey asked participants to identify their agency’s primary activities and the populations they served from a close-ended list. This was followed by the second section, which aimed to capture the degree of collaboration between the respondent agency and other COSACO partners by assessing the frequency of each of the following types of partnership interactions: general, information sharing, joint planning, joint strategic planning, resource sharing, and formal agreement. For each category of interaction, respondents identified the frequency of interactions using a 6-item ordinal scale ranging from *not sure* to *daily*.^12^ The final sections of the survey explored perceptions of partnerships by rating various aspects of partnerships on a 5-point Likert scale, and then provided open-ended questions to assess respondents’ perceived strengths, challenges, and future direction of partnerships, particularly around how these partnerships might optimize visiting volunteer and educational experiences.

### Data analysis

Survey responses were entered into Microsoft Excel 2013 and cleaned, then exported into UCINET 6 (version 6.6) for statistical analysis. From the results of the analysis, network maps and graphs were created using NetDraw and Microsoft Excel 2013. Agencies were grouped by type (academic institution, governmental, non-profit, or healthcare provider), denoted on the maps by different node colours. Agencies were also grouped by location, Dominican Republic (DR) or United States of America (USA), denoted by node shapes.

Averaging and reconstruction are the two ways generally used to reconcile discrepancies in answers between two parties (dyad) regarding their mutual relationship/connection.^14^ In this study, reconstruction was chosen to resolve such discrepancies, meaning that we assumed reciprocity in any reported tie between organizations in the relationship. For example, if Organization A reported a partnership with Organization B, but Organization B did not complete the survey, thus failing to confirm this partnership, we still assumed that partnership existed according to Organization A. Counter-checking was also employed to ensure that no connections were missed because an organization neglected to report the relationship. This process was carried out by verifying the opposing organization’s response regarding the existence of the relationship even if the organization in question did not report it.

Network mapping uses nodes and lines to depict the existence and strength of relationships between individual partners. In addition, the density of nodes and lines portray the level of connection of the network as a whole. We used a map to visualize the described relationships between the agencies in the context of the coalition. To glean additional insight beyond the qualitative network diagram, we used UCINET to calculate network statistics that assessed the proportion of possible connections that exist in the partnership and quantify the position of each organization within the network using statistics of betweenness and betweenness centrality, degree centrality, and network density.^15^

Network density is a measure of the degree of interconnection between agencies. It uses a scale of 0% to 100%, where 0% = no connections between agencies and 100% = every agency is connected with every other agency.^16^ In practical terms, connection to an agency on a specific map means that the two agencies are interacting in that particular area. For example, a connection between two agencies represented as nodes in the sharing of tangible resources map means that the two agencies may be exchanging resources such as medical supplies or educational materials.

Betweenness measures how often an agency is situated on the shortest path between other agencies in the network; agencies with a high measure of betweenness function as a key player in the network in that they act as points of control in communication.^17^ Betweenness centrality measures the extent that an agency plays as a role in the connection of other agencies in the network.^16^ Such an agency would be providing mutual connection to two agencies that would otherwise not have any connections at all. A network achieves a high measure of betweenness centrality when a majority of agencies are connected through mutual connections to a number of key players in the network. For example, with a larger network of 100 agencies, it will be easier for an agency to achieve a betweenness of 5, because there will likely be more potential paths. However, if the network is only the size of 10, there is likely to be less number of paths and therefore a lower likelihood for nodes to be in the path. In this example, a betweenness centrality of 5 would be considered high in a network of 10.

Lastly, degree centrality measures the connectivity of the local network by identifying the most connected agencies, which are defined by nodes with the highest number of direct connections to all other nodes.^18^

The final phase of the study was a thematic analysis of questions on perceived strengths, challenges, and future directions of the partnership. Two authors reviewed responses and quotes and determined emerging thematic trends that would be worthy of further investigation in future studies. Discrepancies around potential inclusion were addressed by a third author and confirmed with the other two reviewers. Summaries of the identified themes were also developed to highlight the perceived strengths and challenges of the coalition. By framing the responses through our objective of investigating the perceived benefits that the coalition provided towards optimizing the outcomes of visiting educational efforts, some of the reported strengths could be interpreted as such benefits.

## Results

### Study participants

All 13 COSACO agencies have some involvement with short-term visiting volunteers and were approached to complete the survey. This included two healthcare provider organizations, five academic institutions, five non-profit organizations, and one governmental agency. Nine of these responding agencies were located in the DR and four were visiting agencies. Of these 13 agencies, nine completed the survey, resulting in a response rate of 69%. Five of these were completed in person during the one-week field visit to DR, two agencies completed the survey interview through Skype; the remaining two were completed via email. Non-respondent members included one academic institution, two non-profit organizations, and one governmental agency, of which three were located in DR and one abroad.

Most COSACO members, including those that sent visiting trainees, were focused on health and development work carried out in settlements established around sugar plantations called *bateyes*; this largely involved the provision of healthcare services and community education. Specific examples ranged from direct primary care services, such as hypertension management and pre-natal care, to broad community programs that supported key determinants of health, such as after school services and workforce development and training.

### Network density and centrality

Our results showed that the COSACO network had moderate connections in general interaction, information sharing, and joint planning, with density scores between 0.4 and 0.2, and weak connections in the categories of joint strategic planning, sharing of tangible resources, and formal agreement, with density scores below 0.2.

Table 1 shows the average betweenness values of the agencies in each of the networks and the average degree centrality of COSACO agencies for each category of interaction. Of note, general interactions and joint planning categories specifically had the highest levels of degree centrality.

**Table 1:** Network statistics

Network	Density	Between-ness	Network Centralization Index	Degree Centrality
General Interactions	46.4%	3.9	24.0%	74.2%
Sharing of Information	27.1%	1.2	5.2%	48.5%
Joint Planning	26.0%	5.2	12.6%	84.9%
Joint Strategic Planning	15.6%	0.6	2.4%	29.6%
Sharing Tangible Resources	9.4%	0.1	0.4%	35.6%
Formal Agreement	17.7%	1.2	5.6%	37.9%

### Network mapping

**Figure 1:**
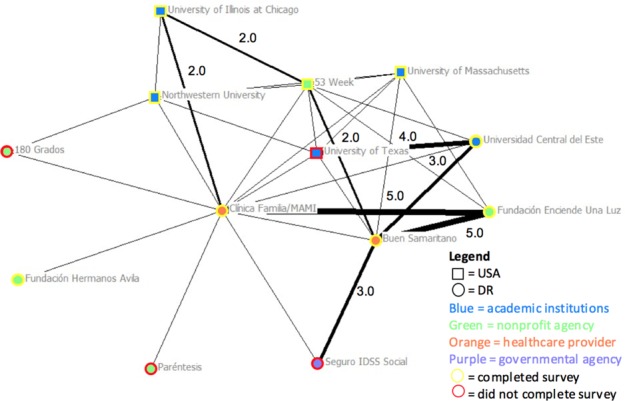
Network based on general interactions among COSACO agencies. Legend: Node shape reflects the geographical location of the agency: circle = DR; square = United States. Node colour reflects the type of agency: blue = academic institution; green = non-profit agency; orange = healthcare provider; purple = governmental agency. Rim colour of nodes reflects survey completion by agency: yellow = completed; red = did not complete. Thickness of the lines reflects the frequency or strength of the connection or interactions: the thicker the line means the higher the frequency of interaction or the stronger the connection.

Figure 1 shows the COSACO network map for the general interactions category. The analysis demonstrated a dense cluster of highly connected agencies. Many of these agencies are connected to each other through one of the DR non-profit healthcare providers, though other DR healthcare providers were also located in the centre of the network.

**Figure 2:**
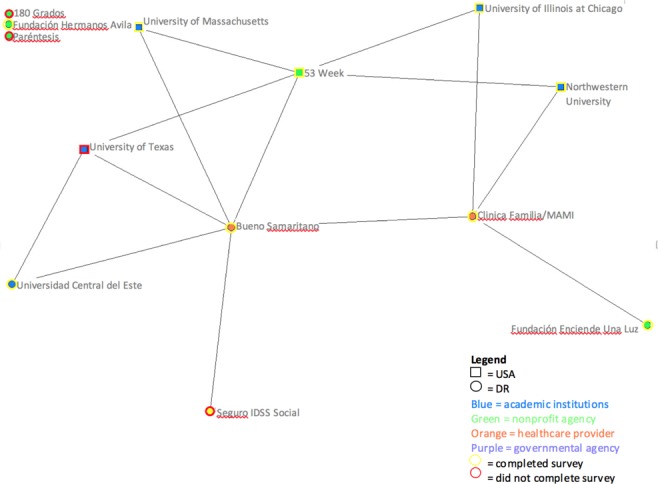
Network based on formal agreements among COSACO agencies. Legend: Each node in the diagram represents an agency. The shape of the node reflects the geographical location of the agency: circle = DR; square = United States. Node colour reflects the type of agency: blue = academic institution; green = non-profit agency; orange = healthcare provider; purple = governmental agency. Rim colour of nodes reflects survey completion by agency: yellow = completed; red = did not complete. Thickness of the lines reflects the frequency or strength of the connection or interactions: the thicker the line means the higher the frequency of interaction or the stronger the connection. Unconnected nodes or isolates: do not have partnerships with other agencies within the network.

Fewer connections were noticed with each progressively connected category of relationships within the network. Of note, the map of formal interactions showed a more typical pattern where DR partners were linked directly to visiting partners. This pattern continued to the map of the resource sharing category, which had the fewest interconnections and the highest number of isolates (agencies with no reported relationships between agencies). In the map of formal interactions, a visiting non-profit organization that has been driving much of the coalition’s work appeared to have the most connections (Figure 2).

### Qualitative analysis of strengths and challenges of partnerships

Survey participants described the strengths of COSACO’s network in relation to three overarching themes around greater enthusiasm, local engagement, and communication (Table 2). The inclusion of agencies from both the DR and USA in this coalition was one identified strength, especially when assessed in relation to educational programming. A representative from one of the visiting academic institutions noted that partnership with locally-based COSACO members “has expanded [students’] understanding of the community [they] serve and given [the institution] ideas and better ways of doing things,” while a local partner’s response commended their academic partners abroad for “[putting] a lot of effort into making locally-led programs succeed.” These responses further affirmed the identified themes as perceived benefits that assisted in the optimizing of outcomes of visiting educational efforts On the other hand, the challenges of the COSACO partnerships were represented by the overarching themes of administration, communication, and engagement (Table 3). Changes in management or administration were a common challenge for many coalition members by various agencies, notably for visiting organizations with a large student leadership component. One such U.S. based institution mentioned that, “[their] leadership changes regularly, which makes it difficult to establish good long-term relationships with individuals in other organizations and build up the trust and respect necessary for good partnership.” Another U.S. based representative noted that they had challenges due to “turnover in partner and internal staff, [which resulted in] lack of continuity of communication.” Of note, the separate theme identified around communication challenges corroborated findings from the network analysis, along with other themes related to lack of resources and the duplication of community work.

**Table 2:** Partnership strengths

Themes in Strengths of Partnerships	Sample Responses
**Communication** Being responsive to partner’s requests General communication Exchanging of ideas Sharing information	“One advantage is [being able] to understand the scope of work of the different organizations working in the bateyes, namely the population which are focused and what kind of work they do in the community” –DR healthcare provider
**Local engagement** Having common goals Maintaining good relationship/friendly interactions Recognizing the need for collaboration	“Local (DR) organizations are also starting to recognize the need to work with other groups to leverage expertise and not just resources” –US nonprofit organization
**Enthusiasm** Recognizing demonstration of effort by partner, leading to increased motivation for both parties	“[Our] partners put a lot of effort into making the programs succeed” –,DR healthcare provider

**Table 3:** Partnership challenges

Themes in Challenges of Partnerships	Sample Responses
**Administration** Challenges in coordination Changes in leadership/internal staff Duplication of community work Lack of funding/resources	“The main challenge has been turnover in partner and internal staff and resultant lack of continuity of communication.” –US academic institution
**Communication** Lack of communication Lack of open information sharing Language barrier	“[Having] few individuals who speak Spanish has also been a limitation.” –US academic institution
**Engagement** Difficulty in aligning missions Not understanding partner’s goals and needs	“There is difficulty in aligning missions.” –DR healthcare provider, said in regards to for-profit vs non-profit organizations

## Discussion

Using network analysis, we characterized the partnership among COSACO agencies by features including general interactions, information sharing, joint planning, joint strategic planning, tangible resource sharing, and formal agreements: this method defined opportunities to address concerns and optimize the conduct of educational programming occurring in La Romana in consultation with leadership.

The network analysis further showed strong measures of betweenness centrality around joint planning (5.2) and general interactions (3.9), which suggests that these two partnership aspects are very dependent on identified key players within the network. Those key players were identified as local healthcare providers and a USA based non-profit organization, termed “boundary spanners,” which are partnership stakeholders with a high number of connections across the network categories mapped out. A reliance on these key players suggests that educators for the visiting institutions must make it a priority to maintain a consistent and open channel of communication with these agencies in order to link their programmatic work to community priorities as identified by COSACO partners.

The general interactions category network map (Figure 1) also confirms that most visiting US-based agencies have some sort of connection to one of two local boundary spanners, resulting most likely from traditional models of global health education and partnership where a visiting institution pairs with a local organization that supports on-the-ground logistics such as transport and interpretation services.^7^ The network maps of formal agreements further confirms the existence of direct linkages between visiting volunteer groups and local partners, which represent these contracted services (Figure 2).

In aiming to move beyond a traditional model towards optimal partnerships that privilege local leadership, COSACO’s strength lies in its centralized communication model as identified by the network analysis that shows that a majority of partnerships are formed through a few largely local boundary spanners. Centralized communication allows the coalition to function more efficiently, thereby enhancing group cohesiveness and increasing the likelihood that educational experiences will be included in community discussion and planning. Literature does suggest, however, that a centralized model is less likely to support accurate group judgments and goal attainment.^19^ Given criticisms of the potential power imbalance that exists between visiting and local stakeholders,^1,3,20,21^ it is incumbent upon the global health educators from visiting institutions to ensure that the work of COSACO remains squarely on aligning the linked partnerships with meeting the community’s health and development goals.

An additional challenge in developing partnerships is limitations in funding and resources that can be committed. This is confirmed in our network analysis where we note that more superficial relationships (such as general interactions) had a higher reported network density, which decreased with each progressively involved category. Corroborated by the qualitative analysis, global health educators would do well to ensure that appropriate funding and resources are committed to the development of local partnerships as part of implementing their educational programming abroad so that the benefits of that knowledge and leadership can ultimately be reflected in curricular decisions.

Of additional interest to educators is the network analysis finding that one visiting U.S. non-profit organization, as a boundary spanner, defies the general trend towards decreased relationships at greater levels of involvement (Figures 1 and 2). This boundary spanner might be able to facilitate the development of resource sharing agreements between visiting and locally-based stakeholders. The implication for global health educators is that pursuing a similar level of partnership through a community coalition could allow them to promote the ethical principle of bi-directionality in programming and resourcing.^22^ This could help share resources in line with identified community needs, such as research data, educational resources (e.g., educational videos, training modules), medical supplies (e.g., medication), and manpower.

As COSACO is a young coalition, future studies are needed to explore if these opportunities have been translated into a robust community health partnership that fosters leadership and guidance from local coalition stakeholders and moves beyond a top-down approach to planning educational experiences abroad.

### Limitations

One limitation was that the inclusion criteria of our study did not specify that the respondent completing the survey should have a leadership role within the agency to try and ensure the responses are truly representative of the agency as a whole. This means that respondents may have been unaware of some of the interagency connections, and unable to accurately report the level of connectedness in partnerships. Although this is perceived to be an important limitation, it was mitigated by reconstruction and counter-checking for reciprocity while conducting the network analysis.

Another limitation was missing data from COSACO members that did not respond. This could have occurred for many reasons; those that did not respond may have been the least connected to the project, or not as well resourced. The latter hypothesis was somewhat demonstrable in the qualitative analysis, where U.S. based organizations were more likely to offer responses than those from the Dominican Republic. This in turn limited the specificity and generalizability of identified themes, though themes identified certainly could form the basis for further exploratory research. Missing data from the network analysis was addressed by assuming reciprocity in the construction of network maps, conceiving it unlikely that a respondent would have reported a partnership with a non-respondent that did not exist.

A final limitation was that no standard definitions were provided to respondents for the categories and questions sought, which may have led to differential interpretations of the questions. However, all respondents were given the opportunity to clarify any questions they may have had, and many of the terms used would not have been dramatically misunderstood or misinterpreted. To mitigate this limitation in future research, explanations and examples for ambiguous terms should be included in the appendix of the survey, especially when translations from one language to another may not be adequate.

### Conclusion

This study has demonstrated the early potential of a community health partnership to improve the fragmentation within existing short-term volunteer efforts being undertaken by coalition partners, and particularly the value that this adds for global health educators seeking to implement study abroad experiences for medical students. The effective use of collaboration and partnership may resolve issues such as duplication of services, and inconsistency in provision of long-term services.^23^ Reaching this goal will require the coalition to privilege the input of local partners, particularly those that are less directly engaged, and ensuring that there is clear elaboration of common goals for the coalition’s work in line with community priorities.

This study secondarily demonstrated the value of network mapping and analysis for educators and trainees to optimize the conduct of short-term medical work abroad and move beyond the traditional single-partner model of global health education and partnership. Network mapping and analysis with identified local educational partners can allow educators to optimize deficient connections and target key efforts towards partnership strengthening that will help to optimize the outcomes and minimize potential harms associated with their activities abroad. Through connecting with local partners with similar interests and goals, educators and trainees conducting medical work abroad will be able to better understand the needs of the local population and periodic follow-up studies of network mapping will allow researchers to track the changes in partnerships as the network grows.

## Appendix A

**Table:** Survey Questions

Types of Question	Questions
Consent to participate in survey	1 I have read and understood the consent form, and I agree to participate in this survey.
General Information (on agency being interviewed)	2 On what target population does your agency focus its efforts? 3 What issue(s) does your agency focus its efforts?
General Interactions	4.a) How often did you interact with the following COSACO agencies?
Information Sharing	4.b)i) How often in the last 12 months did your agency exchange or share information with a specific COSACO agency regarding health related problems or possible solutions for the La Romana residents? 4.b)ii) What type of information did you share with those agencies (i.e., funding opportunities, policy changes, etc.)? 4.b)iii) How many other agencies (excluding the ones previously mentioned) did you share information with in the past 12 months?
Joint Planning	4.c) In the past 12 months, how often did your agency jointly plan, coordinate, or implement an activity, training, event, or program with a specific COSACO agency?
Joint Strategic Planning	4.d)i) Did your agency and a specific COSACO agency jointly negotiate a plan to ensure regular and effective communication between all members? 4.d)ii) Have your agency and a specific COSACO agency jointly negotiated and agreed upon capacity building goals, including the roles and responsibilities of each member?
Resource Sharing	4.e)i) In the last 12 months, did your agency share or exchange tangible resources with a specific COSACO agency? 4.e)ii) Please list the tangible resources your agency shared (not in particular to any agencies).
Formal Agreement	4.f) Did your agency have a formal memorandum of agreement or contract with the specific COSACO agencies regarding the shared resources?
Partnership Satisfaction	5 Rating the following aspects of partnerships on a five-point Likert scale: Allocation of resources Conflict resolution Functioning of governance structure Communication Fairness in financial resources sharing Fairness in allocation of roles and responsibilities Fairness in performance of roles and responsibilities Fairness in providing capacity building opportunities
Open-ended Questions on Partnerships	6 Describe the strengths of partnerships between your agency and other agencies in COSACO in general. 7 Describe two examples of challenges faced in partnerships between your agency and other agencies in general. 8 If your agency had addressed the challenges, describe the steps taken to mitigate them. If not, how does your organization hope to address them in the future? 9 Did your agency explore future directions or new relationship opportunities with other agencies?
